# Testicular Growth and Pubertal Onset in GH-Deficient Children Treated With Growth Hormone: A Retrospective Study

**DOI:** 10.3389/fendo.2021.619895

**Published:** 2021-04-02

**Authors:** Rossella Cannarella, Manuela Caruso, Andrea Crafa, Tiziana Antonia Timpanaro, Manuela Lo Bianco, Santiago Presti, Rosita A. Condorelli, Sandro La Vignera, Aldo E. Calogero

**Affiliations:** ^1^ Endocrinology, Department of Clinical and Experimental Medicine, University of Catania, Catania, Italy; ^2^ Pediatric Endocrinology, Department of Clinical and Experimental Medicine, University of Catania, Catania, Italy

**Keywords:** oligozoospermia, GH deficiency, GHD, IGF1, testicular volume, testicular growth, infertility

## Abstract

The prevalence of idiopathic oligozoospermia has been esteemed as high as 75%. An Italian survey has reported bilateral testicular hypotrophy in 14% of final-year high school students. The search for determinants of testicular growth in childhood is important for the primary prevention of spermatogenic failure. Therefore, this retrospective study aimed to evaluate the testicular growth and pubertal onset in deficient children treated recombinant human growth hormone (rhGH). To accomplish this, the clinical charts of 93 patients with GH deficiency (GHD) were carefully reviewed. Their mean age at the time of diagnosis was 11.2 ± 2.4 years. rhGH was administered for 44.0 ± 22.4 months, and the onset of puberty was recorded after a mean of 25.8 ± 22.4 months from the first rhGH administration. As expected, serum insulin-like growth factor 1 (IGF1) levels increased significantly after treatment. Before rhGH therapy, the Tanner stage was I in 59 out of 70 boys (84.3%), II in 8/70 (11.4%), III in 3/70 (4.3%). No one was on stage IV or V. The mean Tanner stage was 1.19 ± 0.51. At the last visit, the Tanner stage was I in 8/72 boys (11.1%), II in 6/72 (8.3%), III in 6/72 (8.3%), IV in 16/72 (22.2%), and V in 36/72 (50.0%). After a mean of 44.0 ± 22.4 months of rhGH treatment, the mean Tanner stage was 4.05 ± 1.30. Patients treated with rhGH showed a significant testicular volume (TV) growth over time, whereas no growth was observed in age-matched but not yet treated patients, even when the age was compatible with a spontaneous start of puberty. The multivariate regression analysis showed that the duration of treatment and the mean rhGH dose significantly predicted the percentage of TV increase. In contrast, age, serum FSH, and IGF1 levels, and final rhGH dose did not impact TV growth over time. In conclusion, these findings suggest that GH may play a role in testicular growth and pubertal onset, despite the descriptive nature of this study. Further properly designed studies are needed to confirm these findings. This knowledge may be useful to implement the diagnostic-therapeutic algorithm in case of a lack of testicular growth in childhood.

## Introduction

Male infertility represents an increasingly emergent issue in Western countries, since it affects ~7% of the male population (1). However, despite a through diagnostic work-up (including genetic testing), its etiology remains elusive in the vast majority of the cases (2). A longitudinal single center study carried out in 1737 patients with oligozoospermia esteemed the rate of idiopathy as high as 75% (3). Meta-regression data on secular trends of sperm parameters worryingly show that sperm concentration and total sperm count halved in the last forty years. The amount of the annual decrease of total sperm count was 1.6%, overall corresponding to a decline of 59.3% (4). These evidences push toward the urgent need of searching for the causes of apparently idiopathic male infertility.

In recent years, the knowledge on testicular physiology has increased (see 5–7 for review). The Sertoli cells (SCs), the main components of the prepubertal testis, are “nurse cells”, as they release factors enhancing spermatogenesis in the adulthood. Also, SCs constitute the blood-testicular-barrier with the tight-junctions between them, which makes the testicular tubules immunologically silent, so that germ cells are protected by the attack of the immune system. SCs secrete androgen binding protein and anti-Müllerian hormone (AMH), needed for Müllerian ducts regression. Before puberty, SCs are in an immature state during which they actively proliferate and secrete AMH. When puberty starts, SCs switch from an immature to a mature state and lose the ability to mitotically divide. In this phase, they start to release inhibin B and the secretion of AMH declines (5–7).

Every SC provides the niche for the male germline and is able to support the proliferation and differentiation of a definite number of spermatogonia (8, 9). Thus, factors that impact on SC proliferation can likely influence fertility and sperm output in the adulthood. Therefore, at least some of the cases of apparently idiopathic oligozoospermia may be addressed to an abnormal proliferation of SCs in childhood, which entails low testicular volume (TV) in the adulthood. Accordingly, a low TV is associated with low sperm concentration and total sperm count (10). An Italian survey carried out in 3816 final-year high school students found bilateral testicular hypotrophy in up to 14% of cases (11). Hence, to find the factors involved in pre- and peri-pubertal testicular growth and SC proliferation may reasonably be of relevance for the fertility of the tomorrow’s fathers.

Our *in vitro* experience on a model of porcine neonatal SCs suggests that insulin-like growth factor 1 (IGF1) promotes SC proliferation (12). In particular, incubation with IGF1 but not with growth hormone (GH), nor (surprisingly) with follicle-stimulating hormone (FSH), stimulates the proliferation of cultured SCs (12). Despite other data on the experimental animal support the relevance of IGF1 on testicular development and growth (13), the possible implication(s) of this knowledge in the clinical practice is unknown. In particular, the impact of IGF 1 serum levels on testicular growth in childhood has not been fully acknowledged yet. This information could be useful to implement the diagnostic-therapeutic algorithm in case of evidence of poor testicular volumetric growth in childhood.

GH deficiency (GHD) represent a useful clinical model which provides information on the *in-vivo* consequences of the lack of IGF1 and recombinant human GH (rhGH) replacement therapy on testicular growth and pubertal onset in a predefined window of life. Thus, this retrospective study aimed to evaluate the role of GH treatment on testicular growth and pubertal onset in a cohort of GHD children.

## Patients and Methods

### Study Population

This is a retrospective study performed on male GHD children who were diagnosed and followed-up at the Unit of Pediatric Endocrinology, University of Catania (Catania, Italy), from May 2002 to December 2019. Specifically, all children referred for short stature [-2 SDS according to the Italian population-based reference (14)] underwent a complete medical evaluation, including anamnesis, physical examination, blood testing for measurements of complete blood count, fasting glucose, creatinine, aspartate aminotransferase (AST), alanine aminotransferase (ALT), thyroid hormones, and insulin-like growth factor 1 (IGF1). Screening for celiac disease was also accomplished, by the measurement of serum total IgA, transglutaminase IgA, gliadin IgA, and deamidated gliadin IgA. In the case of negative values and no IgA deficiency, the algorithm stopped. In case of IgA deficiency, transglutaminase IgG, gliadin IgG, and deamidated gliadin IgG were measured. Patients with positive values underwent duodenal biopsy for diagnosis confirmation. In those with the suspicion of GHD, plasma GH response to both arginine infusion and glucagon was evaluated and the diagnosis was made when the GH_max_ value at the two GH stimulation tests was <10 µg/l, as for the GH Research Society consensus guidelines (15). All GHD patients received rhGH treatment at a starting dose of 25 μg/kg/day. During follow-ups, dose adjustment was performed within a range of 25–50 μg/kg/day, according to guidelines (15) and in conformity with Note 39 of the Italian Drug Agency (AIFA) (http://www.agenziafarmaco.gov.it/content/nota-39).

Small for gestational age (SGA) patients, those with a head injury, endocrine disorders (hypogonadism, hyperprolactinemia, Cushing syndrome, and hypopituitarism), any form of tumor, exposure to radio- and/or chemotherapy, abnormal FSH levels, systemic diseases (kidney and/or liver diseases), and genetic disorders were excluded.

### Follow-Ups

Patients were followed-up every six months, as the guidelines suggest (15), for auxological measurements and puberty staging. IGF1 and testicular volume were evaluated at each follow-up time. Luteinizing hormone (LH), FSH, total testosterone (TT), and bone age were assessed less frequently when needed (e.g., for monitoring pubertal onset or adverse effects).

Children were followed-up until they reached the near-adult height, or until the end of the study. The treatment was administered until the target height was achieved, independently of the pubertal stage. Pubertal onset was defined for a TV >3 ml by Prader orchidometer. The assessment of puberty was accomplished using the Tanner and Whitehouse staging method (16). The duration of puberty was calculated as the time elapsing between the first measurement of TV >3 ml and the age when the maximum TV was achieved.

### Hormonal Measurements

Hormone evaluation was performed in the central laboratory of the University-Teaching Hospital Policlinico “G. Rodolico-San Marco”, by electrochemiluminescence (ECLIA) (Hitachi-Roche equipment, Cobas 6000, Roche Diagnostics, Indianapolis, IN, USA). Blood was collected at the Pediatrics Endocrinology outpatient clinic, in the morning.

### Statistical Analysis

Results are reported as mean ± SD throughout the study. Outcomes were classified according to rhGH administration. The normality of data distribution was evaluated using the Shapiro-Wilk test. Significant differences between mean TV, gonadotropin, TT, and IGF1 in age-matched treated and still untreated GHD patients were analyzed using the Student *t*-test for independent samples or the Mann-Whitney U test, as appropriate. Multivariate regression analysis was performed for the TV change over time, that is the percentage of TV increase during treatment. It was calculated as the ratio between the TV value at the end of rhGH therapy and TV value before rhGH therapy was started. The variables included in the model were: the length of treatment (number of months during which patients received rhGH therapy), mean IGF1, mean FSH serum levels, calculated from the beginning of puberty to the time when the full testicular development was reached, mean rhGH dosage, final rhGH dosage, age at the end of treatment. SPSS 22.0 for Windows (SPSS Inc., Chicago, USA) and RealStatistics add-on for Excel were used for statistical analysis. The results were considered statistically significant when the *p*-value was lower than 0.05.

### Ethical Approval

This study was conducted at the Division of Endocrinology, Metabolic Diseases and Nutrition of the University-Teaching hospital “G. Rodolico-San Marco”, University of Catania (Catania, Italy). The protocol was approved by the internal Institutional Review Board, and informed written consent was obtained from the parents of each participant after full explanation of the purpose and nature of all procedures used. The study has been conducted in accordance with the principles expressed in the Declaration of Helsinki.

## Results

Medical records of 115 patients were initially evaluated and 93 GHD patients were ultimately included in this study. The remaining 22 were excluded because they had craniofaringioma (n = 3), neuropsychiatric disorder under treatment with risperidone (n = 1), leukemia (n = 1), thalassemia major (n = 3), *short-stature homeobox* (*SHOX*) gene mutations (n = 2), Nijhegen syndrome (n = 1), 1q32.2p43 chromosome deletion (n = 1), Noonan syndrome (n = 1), renal failure (n = 1), medulloblastoma (n = 2), multiple exostoses (n = 1), and abnormalities at brain MRI (n = 2). Three patients with incomplete medical records were also excluded.

At the time of GHD diagnosis, the patients’ mean age was 11.2 ± 2.4 years. The mean rhGH dose initially prescribed was 0.025 ± 0.003 mg/Kg daily; it was modulated at each follow-up according to its efficacy and IGF1 levels. The mean rhGH dose during therapy was 0.026 ± 0.003 mg/Kg daily. rhGH was administered for a mean of 44.0 ± 22.4 months. At the end of the study, patients showed an increase in height and BMI, compared to baseline (**Table 1**). Similarly, the Tanner stage was expectably higher at the end of the study (4.1 ± 1.3) compared to baseline (1.2 ± 0.5), and the onset of puberty was recorded after 25.8 ± 22.4 months from the first rhGH administration.

**Table 1:** Anthropometric and auxological characteristics of the 93 patients with growth hormone (GH) deficiency included in this study at baseline [before the start of recombinant human GH (rhGH) therapy] and at the end of the study.

Parameters	Baseline	End of the study
Age (years)	11.2 ± 2.4	15.4 ± 3.1
Dose of rhGH (mg/kg/daily)	0.024 ± 0.004	0.03 ± 0.01
Height (cm)	127.01 ± 14.88	158.36 ± 11.0
SDS of height	-2.54 ± 0.78	-1.62 ± 0.79
Body mass index (BMI) (Kg/m^2^)	17.93 ± 5.32	20.15 ± 4.60
BMI standard deviation-score (SDS)	-0.40 ± 1.50	-0.77 ± 1.68
Growth velocity (cm/year)	4.18 ± 1.20	5.03 ± 2.55
Growth velocity SDS	-1.72 ± 1.66	2.13 ± 3.47
Tanner stage	1.19 ± 0.51	4.05 ± 1.30

Tanner stage was available for 70 patients at baseline and 72 patients at the end of the study.

**Figure 1:**
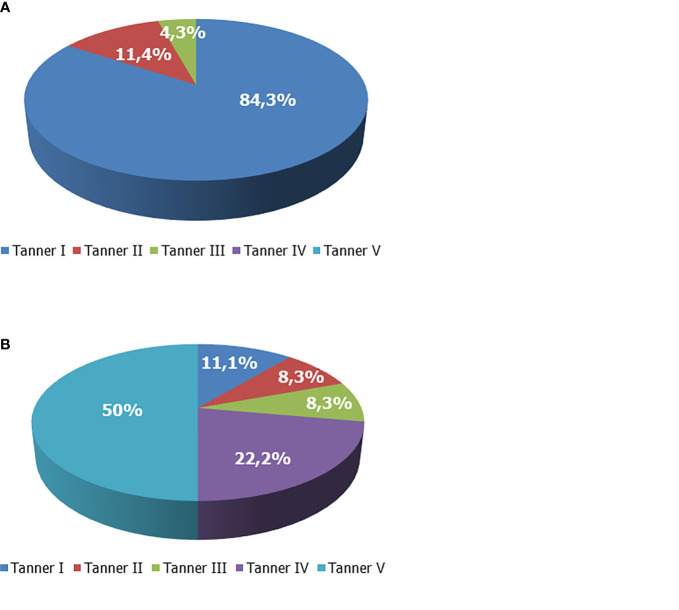
Tanner stage of boys with growth hormone (GH) deficiency. **(A)** Tanner stage at enrollment. **(B)** Tanner stage at the end of the study.

The majority of the patients were pre-pubertal before rhGH was prescribed. In particular, before rhGH therapy, the Tanner stage was I in 59 out of 70 boys, II in 8/70, and III in 3/70. None was on Tanner stage IV or V. At the last visit, the Tanner stage was I in 8 out of 72 boys, II in 6/72, III in 6/72, IV in 16/72, and V in 36/72 (**Figure 1**). Overall, at the end of the study, the treatment was ongoing in 17 patients since they had not already reached the target height. Among them, 10 had a Tanner stage V, 2 a Tanner stage IV, 1 a Tanner stage III, 3 a Tanner stage II, and 1 a Tanner stage I.

**Figure 2:**
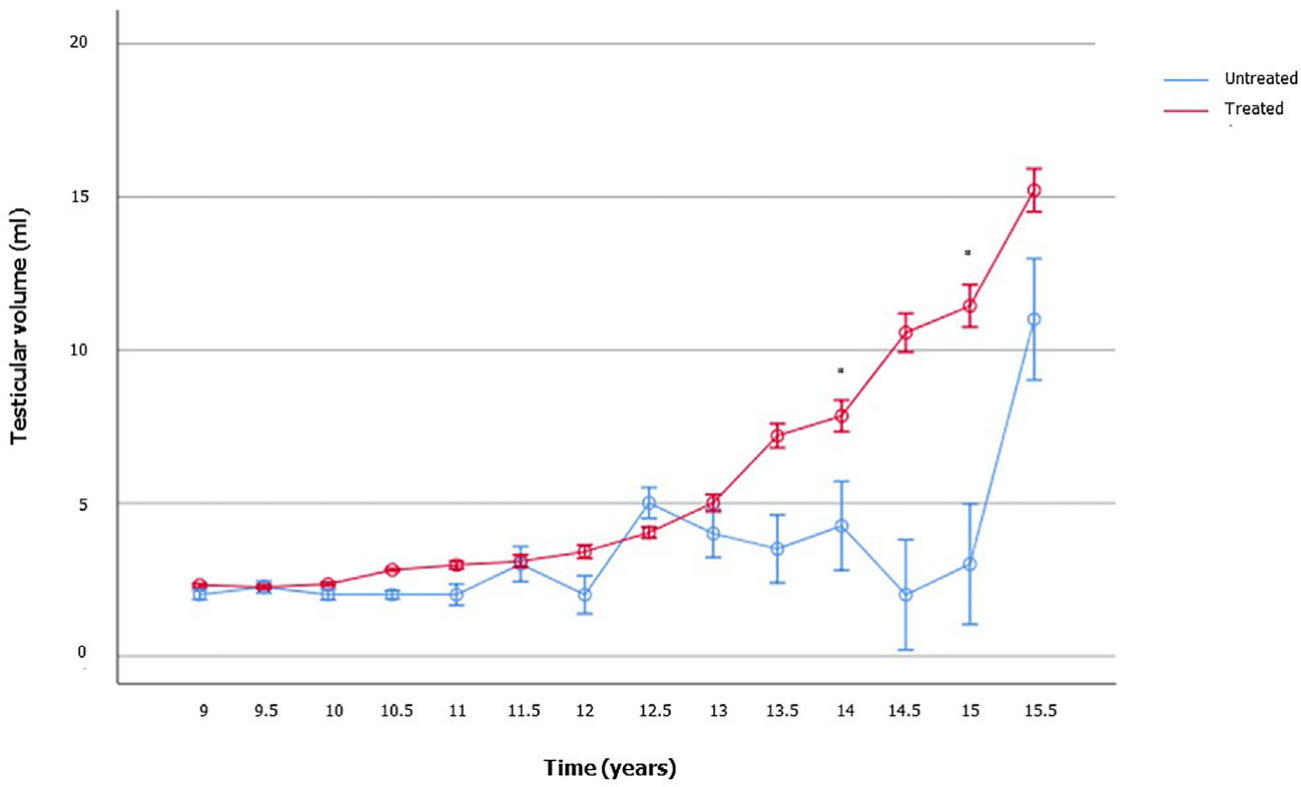
Testicular volume changes over time in boys with growth hormone (GH) deficiency who received recombinant human GH (rhGH) and in aged-matched not yet treated GHD boys. The number of patients available for each time point is reported in the **Supplementary Table 1**.

To appraise the effects of rhGH on the onset of puberty and testicular growth, we evaluated mean IGF1 and TV values at each follow-up visit during GH therapy, and these results were compared with those of the age-matched patients who were not on treatment yet. As showed in **Figure 2**, TV was significantly higher in the treated GHD patients, compared to the age-matched GHD patients who were not on treatment yet. In the patients not already treated, who served as controls, the TV volume remained pre-pubertal, even in those with an age compatible with a spontaneous start of puberty (13-15 years). Only an untreated 15.5 years old patient had a TV of 11 ml (**Figure 2** and **Supplementary Table 1**).

**Figure 3:**
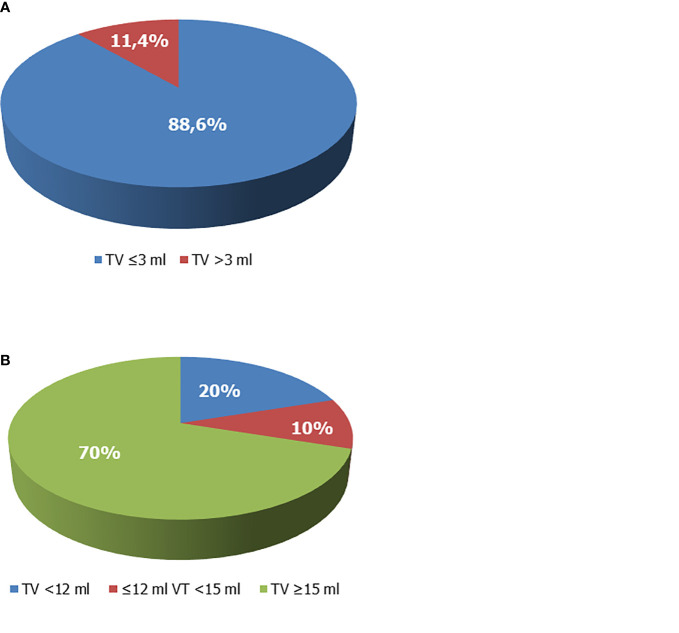
Testicular volume (TV) of boys with growth hormone (GH) deficiency. **(A)** TV at enrollment. Overall, 88.6% of patients whose TV values were available prior to recombinant human GH (rhGH) administration and aged ≥10 years showed TV values ≤3 ml and had a Tanner stage 1. **(B)** TV at the end of rhGH administration. The 70% of patients on rhGH therapy had TV ≥15 ml at the end of therapy, 20% had values <12 ml, compatible with testicular hypotrophy. Ten percent had borderline values (12 ml ≤ TV < 15 ml).

However, baseline TV values were available in 59 patients. Among these, only 5 had a TV >3 ml, which corresponded to 11.4% (5/44) of the patients aged ≥10 years. All the other patients had a TV ≤3 ml (**Figure 3A**). TV at the end of rhGH administration was available in 60 patients, and a full testicular development was reached in 70% of cases (**Figure 3B**). Among patients with TV <12 ml at the last visit, 53.3% (8/15) withdrew from therapy before the age of 16 years.

**Figure 4:**
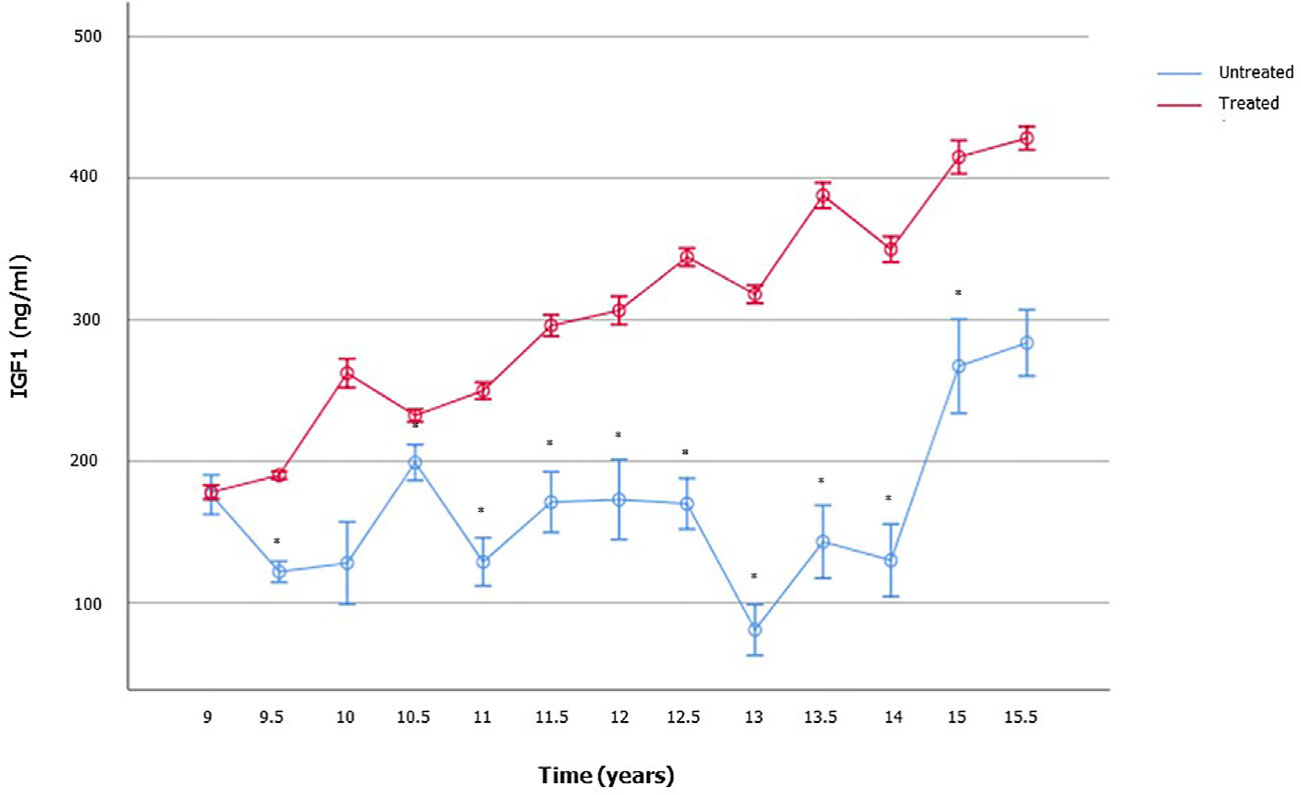
Insulin-like growth factor 1 (IGF1) serum levels in boys with growth hormone (GH) deficiency who received recombinant human GH (rhGH) and in aged-matched not yet treated GHD boys. The number of patients available for each time point is reported in the **Supplementary Table 2**.

Expectably, treated patients showed significantly higher IGF1 levels compared to the age-matched not yet treated ones (**Figure 4**). No difference was found in serum LH, FSH, and TT (**Supplementary Table 2**). These findings suggest the influence of the GH-IGF1 system on TV growth over-time and the onset of puberty.

To evaluate whether the increase in TV was influenced by the duration of rhGH therapy, we built a multivariate regression model, including the length of treatment (number of months during which patients received rhGH therapy), mean IGF1, mean FSH serum levels, calculated from the beginning of puberty to the time when the full testicular development was reached, mean rhGH dosage, final rhGH dosage, and age at the end of treatment. The stepwise procedure showed that the duration of treatment and the mean dose were the only two variables that significantly influenced the percentage of TV increase. By contrast, the other variables were shown not to influence TV growth over time (**Table 2**).

Table 2Stepwise multivariate regression analysis.

**Table:** 

Model with all the variables entered.
	***df***	***SS***	***MS***	***F***	***Significance F***
Regression	6	3613744.43	602290.74	9.9742	0.0000
Regression	6	3613744.43	602290.74	9.9742	0.0000
Residual	29	1751160.33	60384.84		
Total	35	5364904.76

**Table:** Parameters of multivariate regression analysis.

	*β coeff*	*std err*	*t stat*	*p-value*	*Lower 95%*	*Upper 95%*	VIF
Intercept	-1317.0624	816.2324	-1.6136	0.1174	-2986.4452	352.3202	–
Mean IGF1	0.0059	0.3976	0.0148	0.9883	-0.8073	0.8191	1.242
Mean FSH	-19.8334	23.2197	-0.8542	0.4000	-67.3230	27.6562	1.61
Age	28.3438	38.5955	0.7344	0,4686	-50.5928	107.2805	1.475
Duration of treatment (months)	12.4452	1.9576	6.3573	0.0000	8.4415	16.4490	1.226
Mean rhGH dose	31595.3224	22148.8406	1.4265	0.1644	-13704.1430	76894.7878	4.76
Final rhGH dose	226.7791	17306.4988	0.0131	0.9896	-35168.9852	35622.5434	4.051

**Table:** Model after removal of variables with p>0.05.

	*df*	*SS*	*MS*	*F*	*Significance F*
Regression	2	3536761,7	1768381	31,92123	0.0000
Residual	33	1828143,1	55398,27		
Total	35	5364904,8

**Table:** Parameters after Stepwise procedure.

	*β coeff*	*std err*	*t stat*	*p-value*	*Lower 95%*	*Upper 95%*	VIF
Intercept	-861.5613	363.1699	-2.3723	0.0237	-1600.4360	-122.6865	–
Duration of treatment (months)	12.3217	1.7792	6.9253	0.0000	8.7018	15.9416	1.000
Mean rhGH dose	30447.4389	13889.9927	2.1920	0.0355	2188.0362	58706.8416	1.000

IGF1, insulin-like growth factor 1; FSH, follicle-stimulating hormone; rhGH, recombinant human growth hormone.

## Discussion

This retrospective study was undertaken to evaluate whether treatment with rhGH can influence testicular growth and the onset of puberty in a cohort of patients with GHD, the vast majority of whom were pre-pubertal. We found that only the patients receiving rhGH had a progressive increase of the TV over time, whereas still untreated patients did not show any significant TV increase. However, the final TV of the patients treated with rhGH remained lower than the normative values reported in healthy children and adolescents (17). Moreover, TV growth was influenced by the duration of rhGH treatment. Limitedly to the retrospective design of this study, these results suggest a role of GH (and of IGF1) in testicular growth and pubertal onset. On note, the treatment was ongoing in 17 patients at the end of the study, since they had not yet reached the target height. However, the majority of them (10/17) had a Tanner stage V and only 1 was prepubertal (Tanner stage I). Hence, the results were not influenced by the ongoing treatment.

These findings are in agreement with those of the previous literature. However, few studies have assessed the effects of rhGH administration on the onset of puberty and testicular health in the past because this aspect has poorly been explored in GHD. Nevertheless, pubertal delay is a common finding in GHD patients, which has led to treatment with oxandrolone or T in the past (18). The onset of puberty has been reported to occur after 19.0 ± 3.5 months after rhGH therapy initiation and no other specific treatments, in a cohort of eight GHD children. Interestingly, no abnormality of conventional sperm parameters nor TV was found in these patients (19). Subsequently, Bertelloni and colleagues reported low TV and hypergonadotropic hypogonadism in four male patients with non-GHD-dependent short stature treated with rhGH (20). These discrepant data prompted other research groups to assess whether rhGH treatment could be deleterious for the testicular function. Leschek and colleagues designed a randomized, double-blind, placebo-controlled trial to evaluate the effect of rhGH administration on pre-pubertal or early pubertal male patients with non-GH-deficient short stature. The results of this study showed that GH treatment did not have a detrimental effect on testicular function and pubertal onset or pace, as no difference of pubertal onset age or final TV was found compared with placebo (21). Similar findings were reported in a cohort of 84 GHD patients and 27 boys with idiopathic short stature (22). Also, in a cohort of 107 patients (79 with GHD and 28 with idiopathic short stature) treated with rhGH, no difference was found in final TV when compared with that of the reference population, thus confirming that rhGH administration does not impact negatively on testicular volume progression (23). A subsequent study carried out in eight patients with non-GH-deficient short stature (constitutional delay of puberty or idiopathic short stature) showed no adverse effect of rhGH therapy on final TV, gonadotropins, and sperm conventional parameters (24). Finally, in the last prospective, randomized, controlled study on 124 non-GHD-related short children (91 male) randomized in rhGH treatment or placebo, rhGH treatment reported increasing TV (25).

Altogether, these data suggest 1) no detrimental effect of rhGH administration on testicular function in childhood; 2) that rhGH administration may enhance testicular growth in GHD patients that likely occurs *via* an IGF1-mediated mechanism. This hypothesis has been recently suggested also by other authors (26). Indeed, by observing the physiological elevated (acromegalic) levels of IGF1 during puberty in both sexes, IGF1 has been supposed to promote the development of sexual organs and gonads (26). Moreover, IGF1 receptor (IGF1R) has been identified in gonadotropin hormone-releasing hormone (GnRH) neurons of mice, and mice with a selective knock-out of the *IGF1R* gene in the GnRH neurons show marked delay of puberty (26). Hence, IGF1 may act with both a peripheral mechanism – by enhancing SC proliferation, and with a central one – by inducing the firing of the GnRH neurons.

On this account, IGF1 may be suggested as a diagnostic target (with potential therapeutic implications) for cases with poor testicular volumetric growth in childhood. Its serum levels are known to augment with the increase of TV in healthy children (27). Several previous studies have tried to understand if rhGH treatment may somehow improve sperm parameters in adulthood (28–30) or whether a relationship between IGF1 serum levels in adulthood and sperm parameters do exist (31). The results of these studies showed no effect, and, therefore, the idea that the GH-IGF1 may play a role in the reproductive system was abandoned. However, those studies focused on the adult testis, which is made of mature SCs that are unable to proliferate. The physiology of the childhood testis is somewhat different as SCs actively proliferate with an IGF1-dependent mechanism (12). Testicular growth and function in childhood are not frequently investigated by pediatricians. The evaluation of testicular function in prepubertal children may be really important to early recognize markers of testicular tubulopathy and to prevent the onset of apparently idiopathic (and irreversible) oligozoospermia in adulthood (32). In fact, a poor SC proliferation in childhood could lead to a reduced SC number in adulthood, which will support the proliferation and differentiation of a lower number of germ cell, in turn leading to irreversible (since SCs can no more divide) oligozoospermia (8, 9). In this scenario, the measurement of IGF1 may be suggested in children with poor testicular volumetric growth.

We are aware of some of the limitations of the present study. The reasons for cautiously interpreting our results include the retrospective design, which limits the strength of the findings, as well as the lack of an appropriate control group made of untreated GHD patients. However, ethical issues do not allow overcoming this last aspect, since it is not possible to deny rhGH prescription to GHD children. Moreover, the study lacks entirely or in part of some parameters. For example, IGF1 SDS or GH_AUC_ were not available in all children included in the study; also, information on the Tanner stage was available in 70 boys before they were prescribed rhGH and in 72 at the last visit, whereas data on TV were found in 59 boys before therapy and 60 afterward. Finally, not all patients had completed the rhGH therapy at the last visit.

In conclusion, according to the scanty previous literature, a role for GH treatment in testicular growth and pubertal onset in GHD children cannot be excluded. Due to the high prevalence of apparently idiopathic oligozoospermia, the assessment of testicular growth and function in childhood is of importance to prevent the onset of male infertility in adulthood. Longitudinal studies are needed to understand whether a poor testicular volumetric growth may underlie borderline-low IGF1 serum levels in otherwise healthy children. This knowledge may implement the diagnostic-therapeutic algorithm in case of evidence of poor testicular volumetric growth in childhood and could be used for the primary prevention of infertility.

## Data Availability Statement

The raw data supporting the conclusions of this article will be made available by the authors, without undue reservation.

## Author Contributions

RC conceived the study, analyzed the data, and wrote the paper. MC conceived the study and revised the paper. ACr, MB, and SP collected the data. RAC and SV revised the paper. ACa conceived the study and revised the paper. All authors contributed to the article and approved the submitted version.

## Conflict of Interest

The authors declare that the research was conducted in the absence of any commercial or financial relationships that could be construed as a potential conflict of interest.

## References

[1] Cannarella R Condorelli RA Mongioì LM Barbagallo F Calogero AE La Vignera S. Effects of the selective estrogen receptor modulators for the treatment of male infertility: a systematic review and meta-analysis. Expert Opin Pharmacother (2019) 20(12):1517–25. 10.1080/14656566.2019.1615057 31120775

[2] Tüttelmann F Ruckert C Röpke A. Disorders of spermatogenesis: Perspectives for novel genetic diagnostics after 20 years of unchanged routine. Med Genet (2018) 30(1):2–20. 10.1007/s11825-018-0181-7 PMC5838132 29527098

[3] Punab M Poolamets O Paju P Vihljajev V Pomm K Ladva R. Causes of male infertility: a 9-year prospective monocentre study on 1737 patients with reduced total sperm counts. Hum Reprod (2017) 32(1):18–31. 10.1093/humrep/dew284 27864361 PMC5165077

[4] Levine H Jørgensen N Martino-Andrade A Mendiola J Weksler-Derri D Mindlis I. Temporal trends in sperm count: a systematic review and meta-regression analysis. Hum Reprod Update (2017) 23(6):646–59. 10.1093/humupd/dmx022 PMC6455044 28981654

[5] Edelsztein NY Grinspon RP Schteingart HF Rey RA. Anti-Müllerian hormone as a marker of steroid and gonadotropin action in the testis of children and adolescents with disorders of the gonadal axis. Int J Pediatr Endocrinol (2016) 2016:20. 10.1186/s13633-016-0038-2 27799946 PMC5084469

[6] Condorelli RA Cannarella R Calogero AE La Vignera S. Evaluation of testicular function in prepubertal children. Endocrin (2018) 62(2):274–80. 10.1007/s12020-018-1670-9 29982874

[7] La Vignera S Cannarella R Condorelli RA Calogero AE. Disorders of Puberty: Endocrinology of the Pre-Pubertal Testis. J Clin Med (2020) 9(3):780. 10.3390/jcm9030780 PMC7141315 32182985

[8] Orth JM Gunsalus GL Lamperti AA. Evidence from Sertoli cell-depleted rats indicates that spermatid number in adults depends on numbers of Sertoli cells produced during perinatal development. Endocrinology (1988) 122(3):787–94. 10.1210/endo-122-3-787 3125042

[9] Orth JM. Cell biology of testicular development in the fetus and neonate. In: Desjardins C Ewing LL, editors. Cell and molecular biology of the testis. Oxford: Oxford University Press (1993). p. 3–42.

[10] Condorelli R Calogero AE La Vignera S. Relationship between Testicular Volume and Conventional or Nonconventional Sperm Parameters. Int J Endocrinol (2013) 2013:145792. 10.1155/2013/145792 24089610 PMC3780703

[11] Gianfrilli D Ferlin A Isidori AM Garolla A Maggi M Pivonello R. ‘Amico-Andrologo’ Study Group. Risk behaviours and alcohol in adolescence are negatively associated with testicular volume: results from the Amico-Andrologo survey. Andrology (2019) 7(6):769–77. 10.1111/andr.12659 31187607

[12] Cannarella R Mancuso F Condorelli RA Arato I Mongioì LM Giacone F. Effects of GH and IGF1 on Basal and FSH-Modulated Porcine Sertoli Cells In-Vitro. J Clin Med (2019) 8(6):811. 10.3390/jcm8060811 PMC6617362 31174315

[13] Cannarella R Condorelli RA La Vignera S Calogero AE. Effects of the insulin-like growth factor system on testicular differentiation and function: a review of the literature. Andrology (2018) 6(1):3–9. 10.1111/andr.12444 29195026

[14] Cacciari E Milani S Balsamo A Spada E Bona G Cavallo L. Italian cross-sectional growth charts for height, weight and BMI (2 to 20 yr). J Endocrinol Invest (2006) 29(7):581–93. 10.1007/BF03344156 16957405

[15] Growth Hormone Research Society. Consensus guidelines for the diagnosis and treatment of growth hormone (GH) deficiency in childhood and adolescence: summary statement of the GH Research Society. GH Research Society. J Clin Endocrinol Metab (2000) 85(11):3990–3. 10.1210/jcem.85.11.6984 11095419

[16] Tanner JM Whitehouse RH. Clinical longitudinal standards for height, weight, height velocity, weight velocity, and stages of puberty. Arch Dis Child (1976) 51(3):170–9. 10.1136/adc.51.3.170 PMC1545912 952550

[17] Goede J Hack WW Sijstermans K van der Voort-Doedens LM Van der Ploeg T Meij-de Vries A. Normative values for testicular volume measured by ultrasonography in a normal population from infancy to adolescence. Horm Res Paediatr (2011) 76(1):56–64. 10.1159/000326057 21464560

[18] Albanese A Stanhope R. Treatment of growth delay in boys with isolated growth hormone deficiency. Eur J Endocrinol (1994) 130(1):65–9. 10.1530/eje.0.1300065 8124480

[19] Tatò L Zamboni G Antoniazzi F Piubello G. Gonadal function and response to growth hormone (GH) in boys with isolated GH deficiency and to GH and gonadotropins in boys with multiple pituitary hormone deficiencies. FertilSteril (1996) 65(4):830–4. 10.1016/S0015-0282(16)58222-3 8654647

[20] Bertelloni S Baroncelli GI Viacava P Massimetti M Simi P Saggese G. Can growth hormone treatment in boys without growth hormone deficiency impair testicular function? J Pediatr (1999) 135(3):367–70. 10.1016/S0022-3476(99)70136-8 10484805

[21] Leschek EW Troendle JF Yanovski JA Rose SR Bernstein DB Cutler GB Jr. Effect of growth hormone treatment on testicular function, puberty, and adrenarche in boys with non-growth hormone-deficient short stature: a randomized, double-blind, placebo-controlled trial. J Pediatr (2001) 138(3):406–10. 10.1067/mpd.2001.111332 11241051

[22] Ankarberg-Lindgren C Norjavaara E AlbertssonWikland K. Short boys treated with growth hormone show normal progression of testicular size and achieve normal serum testosterone concentrations. Eur J Endocrinol (2002) 146(5):681–5. 10.1530/eje.0.1460681 11980624

[23] Lindgren AC Chatelain P Lindberg A Price DA Ranke MB Reiter EO. Normal progression of testicular size in boys with idiopathic short stature and isolated growth hormone deficiency treated with growth hormone: experience from the KIGS. Horm Res (2002) 58(2):83–7. 10.1159/000064658 12207167

[24] Radicioni AF Paris E De Marco E Anzuini A Gandini L Lenzi A. Testicular function in boys previously treated with recombinant-human growth hormone for non-growth hormone-deficient short stature. J Endocrinol Invest (2007) 30(11):931–6. 10.1007/BF03349240 18250614

[25] Albin AK Ankarberg-Lindgren C Tuvemo T Jonsson B Albertsson-Wikland K Ritzén EM . Does growth hormone treatment influence pubertal development in short children? Horm Res Paediatr (2011) 76(4):262–72. 10.1159/000329743 21921571

[26] Juul A Skakkebæk NE. Why Do Normal Children Have Acromegalic Levels of IGF-I During Puberty? J Clin Endocrinol Metab (2019) 104(7):2770–6. 10.1210/jc.2018-02099 30840065

[27] Juul A Bang P Hertel NT Main K Dalgaard P Jørgensen K. Serum insulin-like growth factor-I in 1030 healthy children, adolescents, and adults: relation to age, sex, stage of puberty, testicular size, and body mass index. J Clin Endocrinol Metab (1994) 78(3):744–52. 10.1210/jc.78.3.744 8126152

[28] Radicioni A Paris E Dondero F Bonifacio V Isidori A. Recombinant-growth hormone (rec-hGH) therapy in infertile men with idiopathic oligozoospermia. Acta Eur Fertil (1994) 25(5):311–7. 7660721

[29] Lee KO Ng SC Lee PS Bongso AT Taylor EA Lin TK. Effect of growth hormone therapy in men with severe idiopathic oligozoospermia. Eur J Endocrinol (1995) 132(2):159–62. 10.1530/eje.0.1320159 7858733

[30] Carani C Granata AR De Rosa M Garau C Zarrilli S Paesano L. The effect of chronic treatment with GH on gonadal function in men with isolated GH deficiency. Eur J Endocrinol (1999) 140(3):224–30. 10.1530/eje.0.1400224 10216517

[31] Andreassen M Jensen RB Jørgensen N Juul A. Association between GH receptor polymorphism (exon 3 deletion), serum IGF1, semen quality, and reproductive hormone levels in 838 healthy young men. Eur J Endocrinol (2014) 170(4):555–63. 10.1530/EJE-13-0729 24412931

[32] La Vignera S Condorelli RA Cimino L Cannarella R Giacone F Calogero AE. Early Identification of Isolated Sertoli Cell Dysfunction in Prepubertal and Transition Age: Is It Time? J Clin Med (2019) 8(5):636. 10.3390/jcm8050636 PMC6572413 31075862

