# Supplementary material for: Testicular Growth and Pubertal Onset in GH-Deficient Children Treated With Growth Hormone: A Retrospective Study
Source: Front Endocrinol (Lausanne). 2021 Apr 2;12:619895. doi: 10.3389/fendo.2021.619895 (PMC8050347; doi:10.3389/fendo.2021.619895)
Supplement: Supplementary file 1 [file Table_1.pdf]

Supplementary Table 1. Mean (±standard deviation) testicular volume measured by Prader's orchidometer in treated and untreated boys with growth hormone deficiency (GHD) and in healthy ones.

**Age (years)**	**n**	**GHD patients after the start of therapy (ml)**	**n**	**GHD patients before the start of therapy (ml)**	**n**	**Healthy boys[a] (ml)**
6.5	1	1.0	1	2.0	---	---
7.0	1	2.0	2	2.0 (0.0)	62	2.03 (0.47)
7.5	2	2.25 (0.35)	1	2.0	---	---
8.0	4	2.13 (0.25)	1	1.0	59	2.08 (0.59)
8.5	5	2.20 (0.57)	2	2.25 (1.06)	---	---
9.0	8	2.31(0.51)	4	1.75 (0.50)	53	2.31 (1.02)
9.5	8	2.25 (0.65)	2	2.13 (0.8)	---	---
10.0	10	2.25 (0.53)	4	2.25 (0.65)	49	2.67 (0.98)
10.5	13	2.63 (0.55)	4	2.0 (0.0)*	---	---
11.0	16	2.72 (0.94)	8	2.34 (0.58)	60	3.48 (1.97)
11.5	21	3.10 (1.30)	3	3.66 (1.60)	---	---
12.0	27	3.65 (1.88)	7	3.25 (0.84)	55	5.73 (3.63)
12.5	26	4.57 (2.30)	1	5.0	---	---
13.0	32	5.68 (3.48)	4	3.25 (0.64)	47	10.16 (5.99)
13.5	32	7.45 (3.46)	4	4.06 (0.82)	---	---
14.0	40	9.46 (4.67)	4	3.81(1.07)*	35	15 (7.02)
14.5	35	10.80 (5.69)	2	3.25 (1.76)	---	---
15.0	37	13.30 (5.64)	2	4.0 (1.41)*	26	19.04 (5.76)
15.5	37	15.27 (5.96)	1	11.0	---	---
16.0	35	16.46 (5.24)	---	---	31	23.92 (2.8)
16.5	33	17.98 (4.53)	---	---	---	---
17.0	32	18.70 (4.08)	---	---	27	24.63 (3.88)
17.5	31	19.76(3.28)	---	---	---	---
18.0	31	20.08 (3.11)	---	---	23	24.41 (3.83)

Abbreviations: n= number. [a][Horm Res Paediatr 76(1), 56-64]; *$p \leq 0.05$ vs. GHD patients after the start of therapy, Student *t*-test.

Supplementary Table 2. Mean (±standard deviation) FSH, LH, TT, and IGF1 serum levels in treated boys and before the start of therapy with growth hormone deficiency.

Age (years)	n	FSH after treatment (IU/l)	n	FSH before treatment (IU/l)	n	LH after treatment (IU/l)	n	LH before treatment (IU/l)	n	TT after treatment (ng/ml)	n	TT before treatment (ng/ml)	n	IGF1 after treatment (ng/ml)	n	IGF1 before treatment (ng/ml)
6.5	---	---	---	---	---	---	---	---	---	---	---	---	1	120.5	2	69.45 (20.01)
7.0	---	---	----	---	---	---	---	---	---	---	---	---	2	167.30 (43.70)	1	91.0
7.5	---	---	---	---	---	---	---	---	---	---	---	---	3	136.10 (13.30)	2	76.5 (61.52)
8.0	1	1.2	1	0.3	1	0.06	1	0.1	1	0.07	---	---	5	170.12 (47.80)	1	105.0
8.5	---	---	---	---	---	---	---	---	---	---	---	---	5	176.46 (57.24)	3	105.2 (4.88)
9.0	---	---	---	---	---	---	---	---	---	---	---	---	8	188.18 (70.0)	3	135.13 (36.16)
9.5	---	---	---	---	---	---	---	---	---	---	---	---	8	190.09 (37.23)	4	111.25 (55.58)*
10.0	---	---	---	---	---	---	---	---	---	---	---	---	17	241.85 (110.89)	3	127.33 (26.91)
10.5	---	---	---	---	---	---	---	---	---	---	---	---	10	231.0 (57.0)	5	141.12 (61.97)*
11.0	1	0.73	2	0.82 (0.06)	1	0.04	2	0.04 (0.02)	1	0.13	2	0.11 (0.03)	20	245.75 (72.13)	7	164.72 (62.34)*
11.5	2	4.18 (3.50)	---	---	1	0.01	---	---	1	3.86	---	---	18	291.11 (108.28)	3	165.37 (37.83)*
12.0	5	1.35 (0.48)	2	1.39 (0.90)	7	1.21 (1.36)	2	0.34 (0.37)	3	0.13 (0.01)	2	0.80 (0.11)	28	314.25 (145.70)	7	193.33 (49.22)*
12.5	7	1.86 (1.30)	---	---	7	1.40 (1.26)	---	---	8	0.50 (0.87)	---	---	25	353.94 (119.70)	2	180.5 (14.84)*
13.0	8	2.95 (1.51)	3	2.02 (1.19)	8	0.72 (0.30)	2	1.05 (0.35)	8	0.26 (0.27)	1	0.16	31	349.98 (131.25)	5	131.54 (41.46)*
13.5	6	2.37 (1.33)	2	1.08 (0.31)	4	0.72 (0.52)	2	0.36 (0.42)	5	0.54 (0.79)	2	0.31 (0.30)	30	385.61 (128.21)	5	144.24 (23.69)*
14.0	14	1.83 (0.88)	2	2.19 (0.55)	14	0.97 (0.44)	2	0.77 (0.52)	14	0.77 (0.65)	1	0.29	38	423.94 (132.01)	4	142.1 (52.96)*
14.5	9	1.76 (1.38)	---	---	9	1.59 (1.12)	---	---	10	1.19 (1.41)	---	---	39	439.97 (158.72)	---	---
15.0	13	2.58 (2.21)	1	2.16	14	1.29 (0.87)	1	0.69	13	2.62 (2.80)	1	0.22	38	472.57 (143.46)	2	229.1 (53.88)*
15.5	7	2.15 (1.51)	---	---	7	1.52 (1.06)	---	---	7	2.42 (1.84)	---	---	30	424.25 (129.41)	---	---
16.0	9	3.01 (2.55)	---	---	9	1.66 (1.32)	---	---	9	2.90 (2.27)	---	---	30	466.85 (111.21)	---	---
16.5	8	3.29 (1.28)	---	---	8	1.82 (0.79)	---	---	8	3.10 (2.03)	---	---	25	495.43 (141.34)	---	---
17.0	6	4.10 (1.82)	---	---	6	3.03 (1.55)	---	---	6	4.31 (1.18)	---	---	21	496.16 (148.87)	---	---
17.5	2	3.63 (2.84)	---	---	2	2.41 (0.45)	---	---	3	5.22 (0.75)	---	---	13	436.25 (61.63)	---	---
18.0	1	13.63	---	---	1	4.03	---	---	2	5.64 (0.96)	---	---	10	451.86 (123.48)	---	---

Abbreviations: FSH, follicle stimulating hormone; LH, luteinizing hormone; TT, total testosterone; IGF1, insulin-like growth factor 1. $^{*}p \leq 0.05$ vs. Treated GHD patients, Student *t*-test.
